# Supplementary figures and images for: Large-scale map of RNA binding protein interactomes across the mRNA life-cycle
Source: bioRxiv. 2023 Jun 8:2023.06.08.544225. Preprint. [Version 2] doi: 10.1101/2023.06.08.544225 (PMC10274859; doi:10.1101/2023.06.08.544225)

Supplemental Figure 1

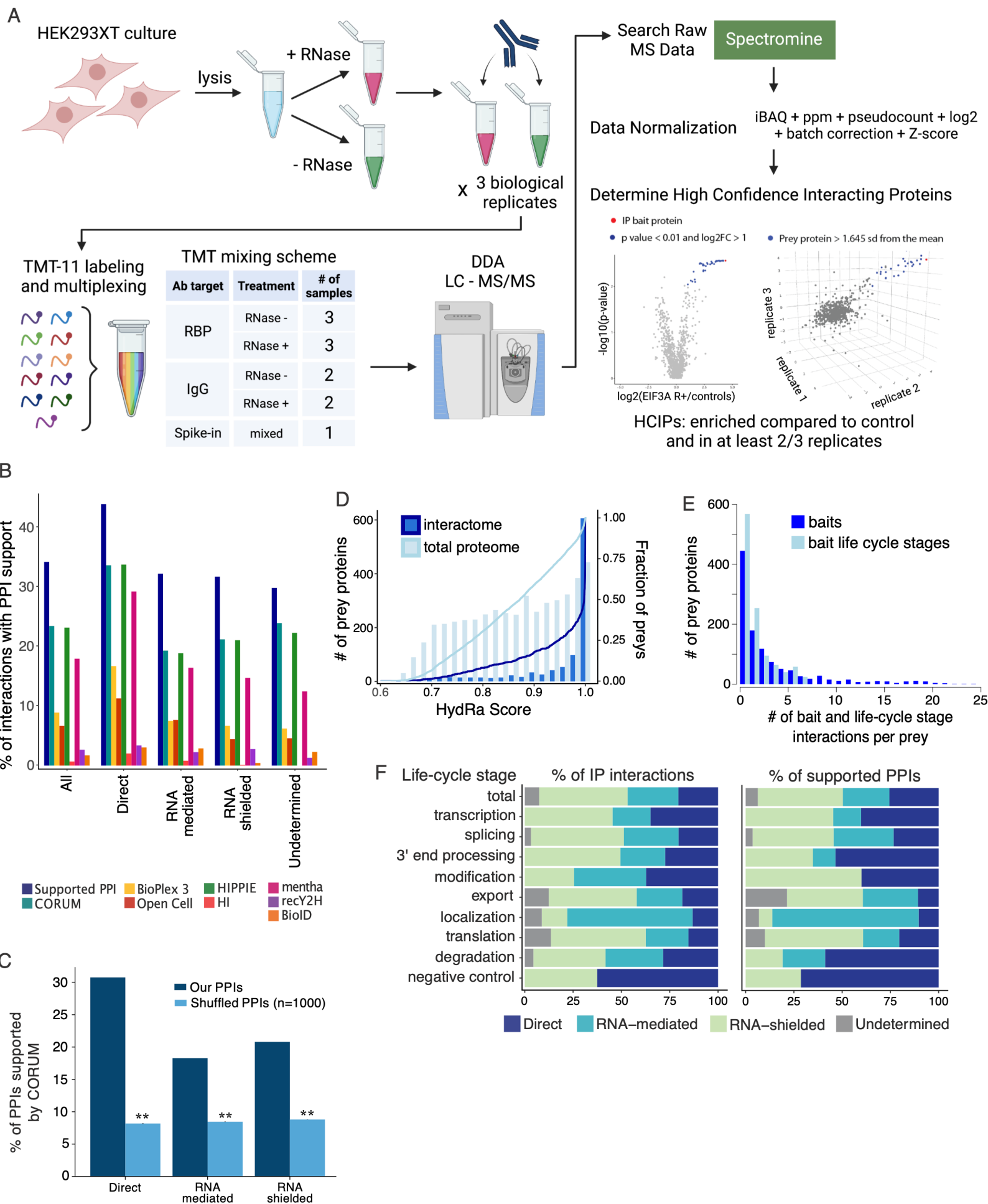

Supplement: Supplement 1 — Supplemental Figure 1. The IP-MS network significantly recapitulates known PPIs across life-cycle stages and interaction types A. Schematic of the experimental workflow. The IP was performed in the presence and absence of RNase using endogenous antibodies followed by LC-MS/MS for each bait. IPs against the baits were performed in triplicate for both with and without RNase conditions and IgG controls were performed in enough replicates in with and without RNase conditions to meet the requirements of the TMT mixing scheme. IP samples were multiplexed into TMT-11 mixes with a spike-in channel made from a mix of all IPs in the dataset, 3 RBP IPs with and without RNase and 2 IgG controls with and without RNase. Raw data was searched using the SpectroMine software and the data was normalized. High confidence interacting proteins were identified as those preys that have normalized intensities greater than 1.645 sd in at least 2 replicates and that were significantly enriched compared to control and failed IPs (see methods for details). B. The percentage of literature supported interactions in the IP-MS network and by interaction type for a number of different databases and previously published large-scale interactomes. C. The distribution of different types of interactions for the baits assigned across the mRNA life-cycle (left), and the distribution of interaction types for those PPIs with literature support (right). D. Percent of CORUM supported interactions in the network compared to randomly shuffled interactions of all the proteins in our network for n = 1000 tests, p-value < 0.001. E. Histograms of the number of baits or unique bait life-cycle stages for the preys in the IP-MS network. F. Prediction of RNA binding for preys in the interactome compared to proteins measured in the total HEK293XT proteome. RNA binding prediction by HydRa scores (greater scores indicate more confidence of RNA binding; score >0.89 indicates predicted RBP). K-S statistic: p-value < 2.2e−16 [file media-1.pdf]

Supplemental Figure 2

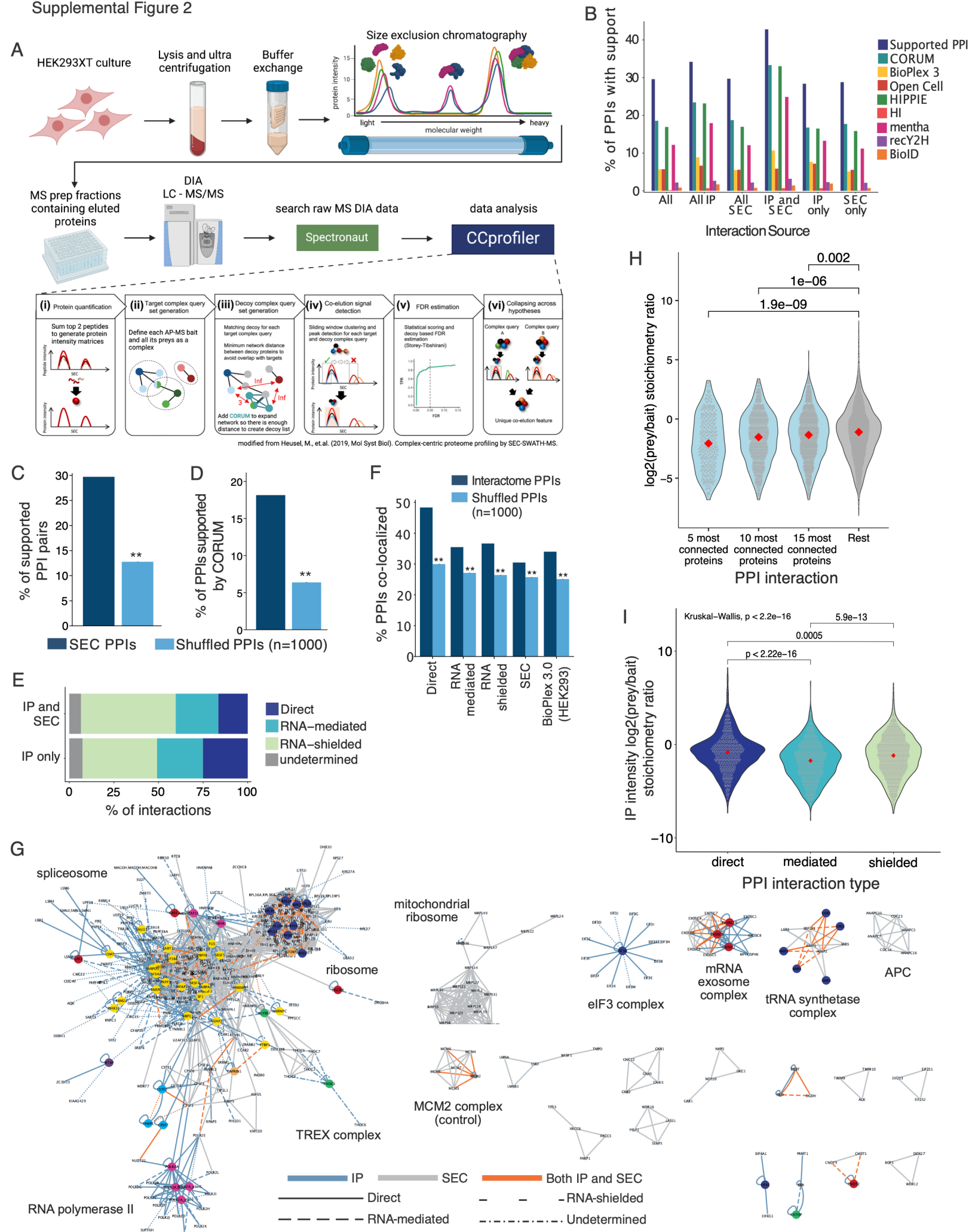

Supplement: Supplement 2 — Supplemental Figure 2. The IP+SEC network has significant levels of co-localization and different interaction stoichiometries between complex hubs and connector proteins A. Schematic of the experimental workflow. Lysates from HEK293XT cells are clarified and buffer exchanged into SEC buffer before being run on a SEC column with fraction collection. Fractions are processed for mass spectrometry and run on LC-MS/MS in DIA mode. Raw data is searched using Spectronaut software and protein co-elution is evaluated by CCprofiler at an FDR of 0.1 and q-value < 0.1 against a dataset of decoy interactions. B. Percent of interactions by interaction type that are supported by SEC-MS and IP-MS compared to those only found in the IP-MS network. C. Percent of supported interactions in the SEC-MS network compared to randomly shuffled interactions of the proteins within our network for n=1000 tests, p-value < 0.001. D. Percent of CORUM supported interactions in the SEC-MS network compared to randomly shuffled interactions for n=1000 tests, p-value < 0.001. E. The percentage of literature supported interactions in the SEC-MS, IP-MS, overlap, and combined networks for a number of different databases and previously published large-scale interactomes. F. Percent of PPIs with fully or partially co-localized annotations in the Human Protein Atlas in the IP+SEC-MS network and in the previously published large-scale Bioplex 3.0 interactome compared to randomly shuffled interactions for n=1000 tests, p-value < 0.001. G. The stoichiometric ratios in the IP-MS data for the 5, 10, and 15 most connected proteins in the network are significantly lower (adjusted p-value Student’s t-test) than the ratios of the rest of the network. Medians are plotted in red. H. Stoichiometric ratios of interactions found in the network. The log2 ratios of the intensities of the prey over bait in the IP-MS data are plotted by interaction type with adjusted Student’s t-test significance values. Medians plotted in re [file media-2.pdf]

Supplemental Figure 3

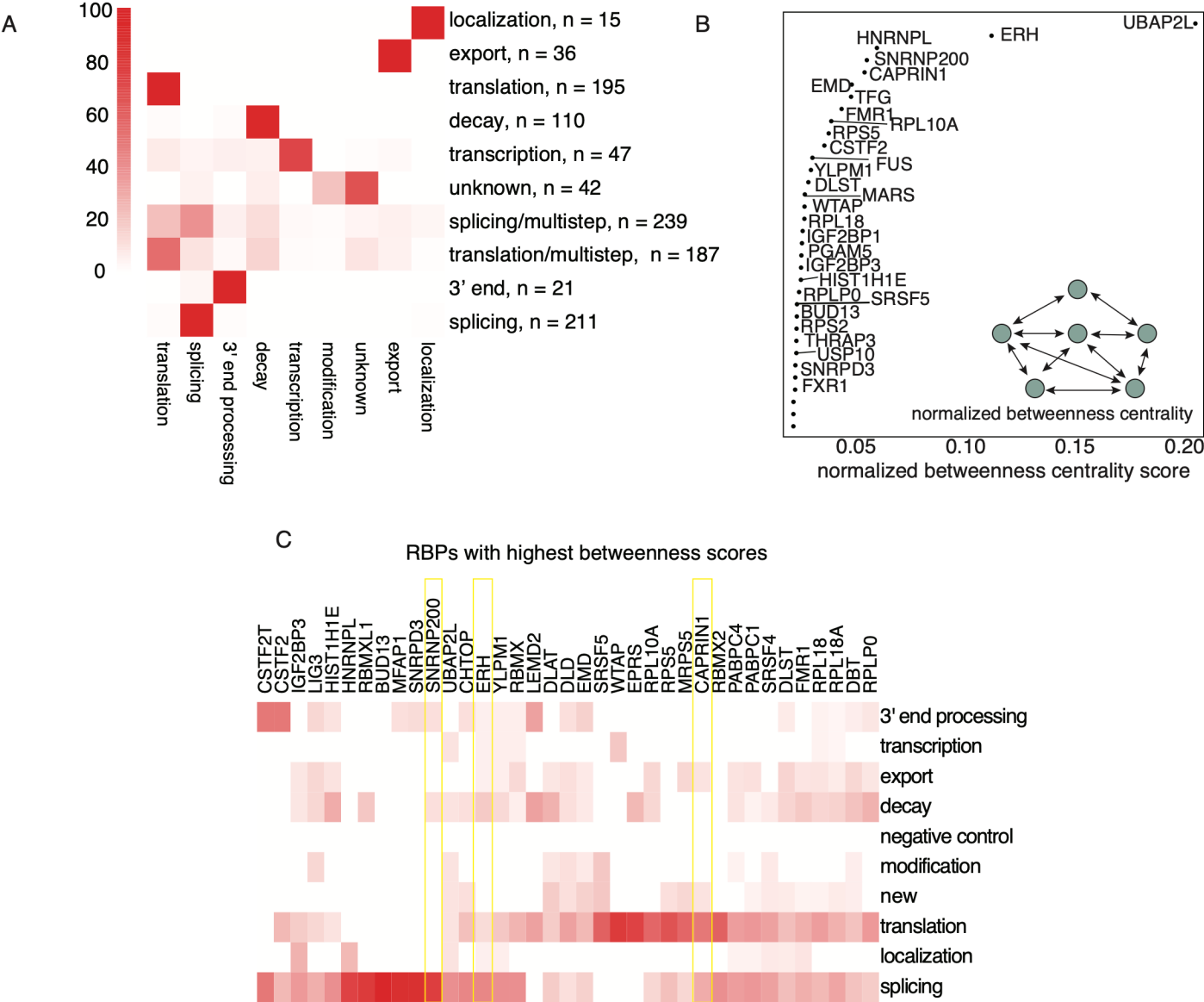

Supplement: Supplement 3 — Supplemental Figure 3. Bait- and Prey-centric analyses link proteins to particular life-cycle steps and complexes A. Heatmap of hierarchical clustering grouping of the distribution of life-cycle steps associated with each prey based on the life-cycle associations of its interacting bait(s). B. Heatmap of correlation between bait-bait interactions. C. Cumulative distribution plot of the traffic flow normalized betweenness centrality scores. The top 3% of RBPs are plotted. D. Subset of Figure 3A plotting the top 30 preys with the highest centrality score. For each prey, the percent of their baits from each life-cycle step is plotted. [file media-3.pdf]

Supplemental Figure 4

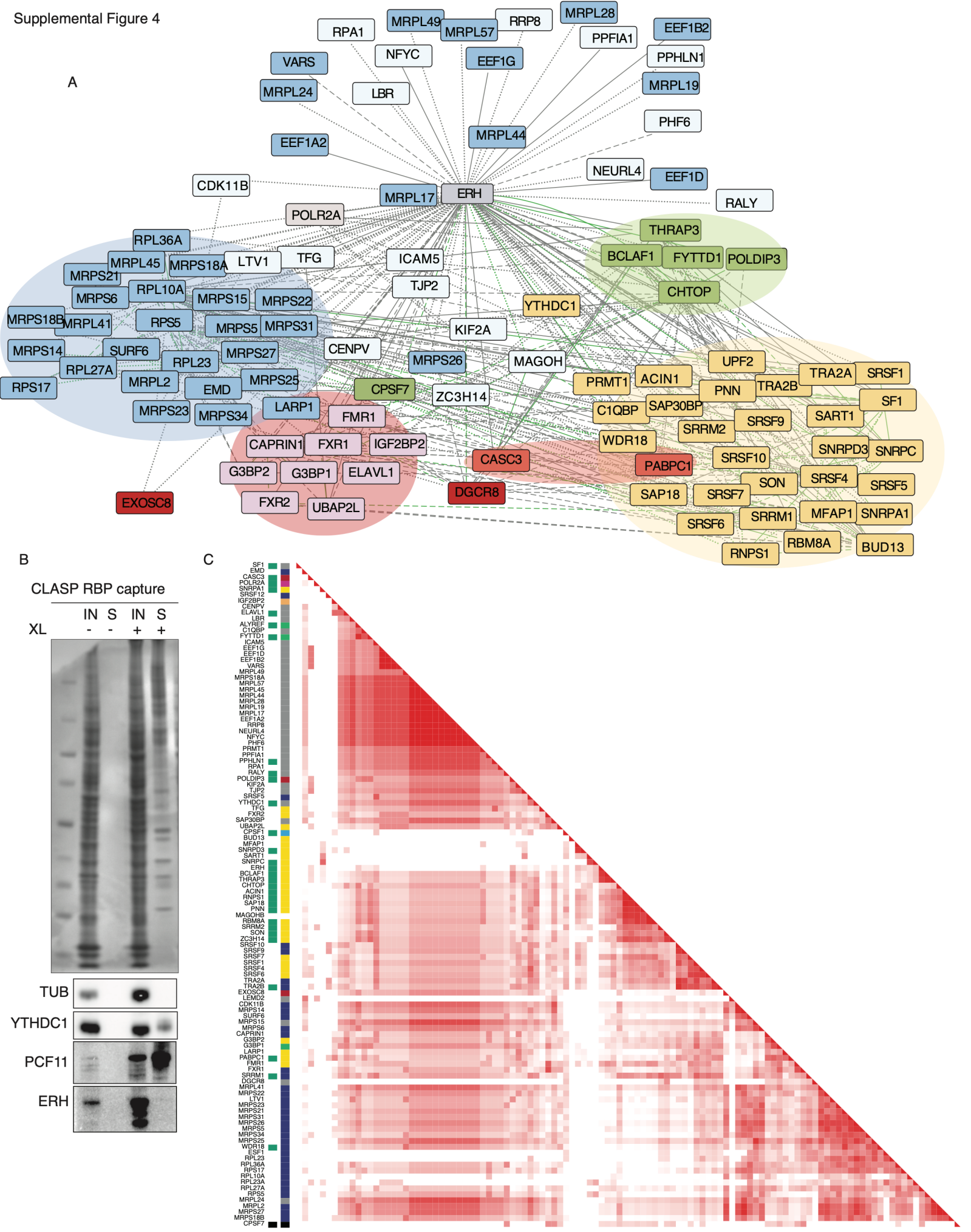

Supplement: Supplement 4 — Supplemental Figure 4. ERH, a non-RBP, associates with nuclear speckles proteins A. Cytoscape plot of all the ERH interactions (including RNA shielded, mediated and direct interactions). Groups of RBPs are colored by GO ontology associated with mRNA life-cycle steps. B. Prey-prey plot of the proteins that interact with ERH. Each protein is colored based on the hierarchical clustering assignment to a particular life-cycle step. Proteins are also colored if they were found to be in nuclear speckles (green). C. Top: Total protein blot of CLASP experiment. Bottom: Immunoblot of the CLASP experiment staining for the RBPs YTHDC1, PCF11 and the negative control protein TUB4A and experimental protein ERH. [file media-4.pdf]

Supplemental Figure 6

A

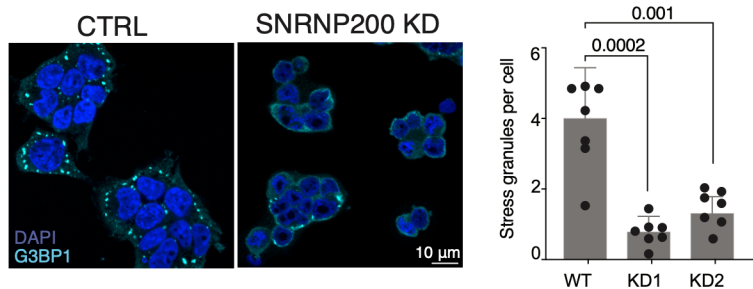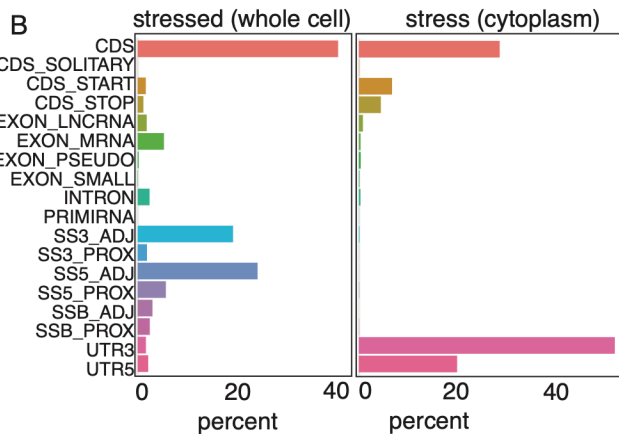

D

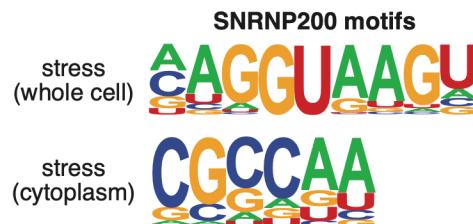

C

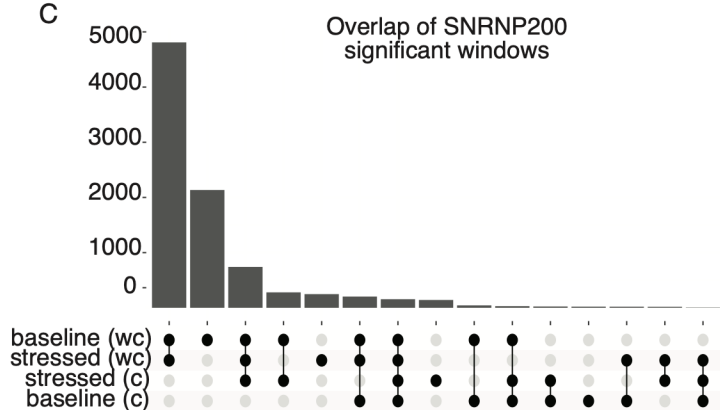

E

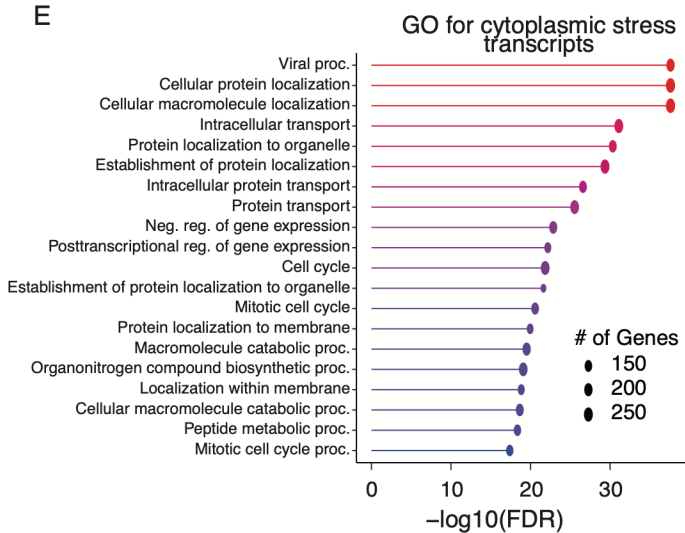

F

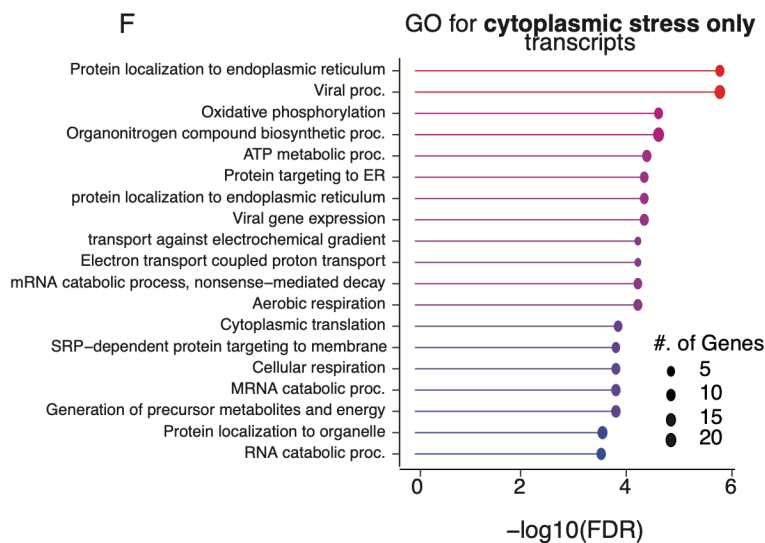

Supplement: Supplement 6 — Supplemental Figure 6. SNRNP200 binds a distinct set of transcripts during stress A. Immunofluroscence images counting the number of stress granules (G3BP1) in each cell in shRNA SNRNP200 knockdown and control. Bargraph of the quantification of the number of stress granules per cell across 10 images. Student’s paired t test was used to calculate if there was a difference in the means. B. Barplot of the percentage of windows that mapped to transcript features in stressed whole-cell versus stressed cytoplasmic SNRNP200 eCLIP. C. Upset plot of significant (p-value < 0.001 and enrichment > 3) windows from SNRNP200 eCLIP samples from whole-cell and cytoplasmic SNRNP200 at baseline and stressed conditions. D. Most significant HOMER motif analysis of stressed whole-cell and cytoplasmic SNRNP200. E. GO analysis for transcripts bound by cytoplasmic SNRNP200 during stress. F. GO analysis for transcripts that are distinctly bound by SNRNP200 in the cytoplasm during stress. [file media-6.pdf]
