# Supplementary material for: Large-scale map of RNA binding protein interactomes across the mRNA life-cycle
Source: bioRxiv. 2023 Jun 8:2023.06.08.544225. Preprint. [Version 2] doi: 10.1101/2023.06.08.544225 (PMC10274859; doi:10.1101/2023.06.08.544225)

Supplemental Figure 5

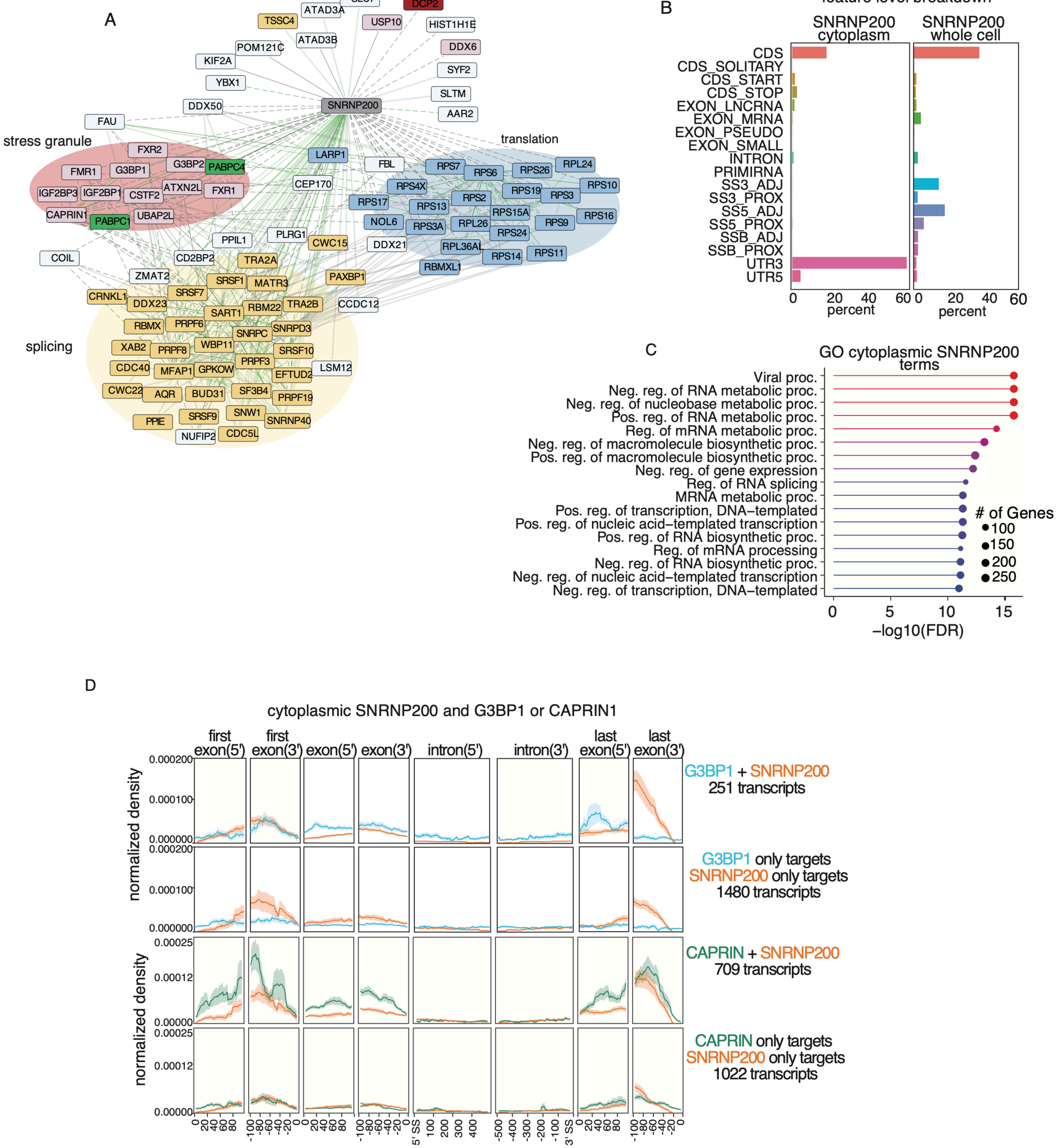

Supplement: Supplement 5 — Supplemental Figure 5. SNRNP200 interacts and co-binds transcripts with stress granule proteins CAPRIN and G3BP1 A. Full cytoscape network of SNRNP200 interactions. Proteins are grouped based on GO ontology terms. B. Barplot of the percentage of SNRNP200 windows that mapped to specific transcript features (5’UTR, CDS, 3’UTR etc.) in the whole-cell eCLIP vs the cytoplasmic eCLIP. C. GO terms for the transcripts bound by SNRNP200 in the cytoplasmic fraction. Terms are ordered by FDR values. D. Metadensity plots showing the binding profiles of G3BP1 (blue) or CAPRIN (green) and SNRNP200 (orange) for transcripts that are known to be bound by both RBPs or exclusively by cytoplasmic SNRNP200. [file media-5.pdf]
